# Chlorido[3,3′-dibutyl-5,5′-(pyridine-2,6-di­yl)dipyrazol-1-ido]gold(III)

**DOI:** 10.1107/S1600536809050995

**Published:** 2009-12-16

**Authors:** A. Stephen K. Hashmi, Christian Lothschütz, Frank Rominger

**Affiliations:** a Organisch-Chemisches Institut, Ruprecht-Karls Universität Heidelberg, Im Neuenheimer Feld 270, 69120 Heidelberg, Germany

## Abstract

The Au atom in the *C*2-symmetric pincer-type title complex, [AuCl(C_19_H_23_N_5_)], is in the +3 oxidation state. The ligand is composed of one pyridine unit and two *n*-butyl-substituted pyrazoles (pyrz). Both pyrazoles are deprotonated, thus forming a neutral compound. To the best of our knowledge, this is the first Au^III^–bis­pyrazolate complex. According to the special geometry in the *N*,*N*′,*N*′′-tridentate ligand, containing two five-membered heterocycles, the complex deviates from an ideal square-planar coordination geometry; the N_pyrz_—Au—N_pyrz_ angle is 160.8 (3)°, indicating a distortion of nearly 20°.

## Related literature

For the importance of gold catalysis, see: Hashmi & Hutchings (2006*a* ▶,*b* ▶); Hashmi (2007 ▶). For the role of the gold(I) oxidation state, see: Ito *et al.* (1986 ▶) and for the use of gold(III) pre-catalysts, see: Hashmi *et al.* (2004*a* ▶,*b* ▶).
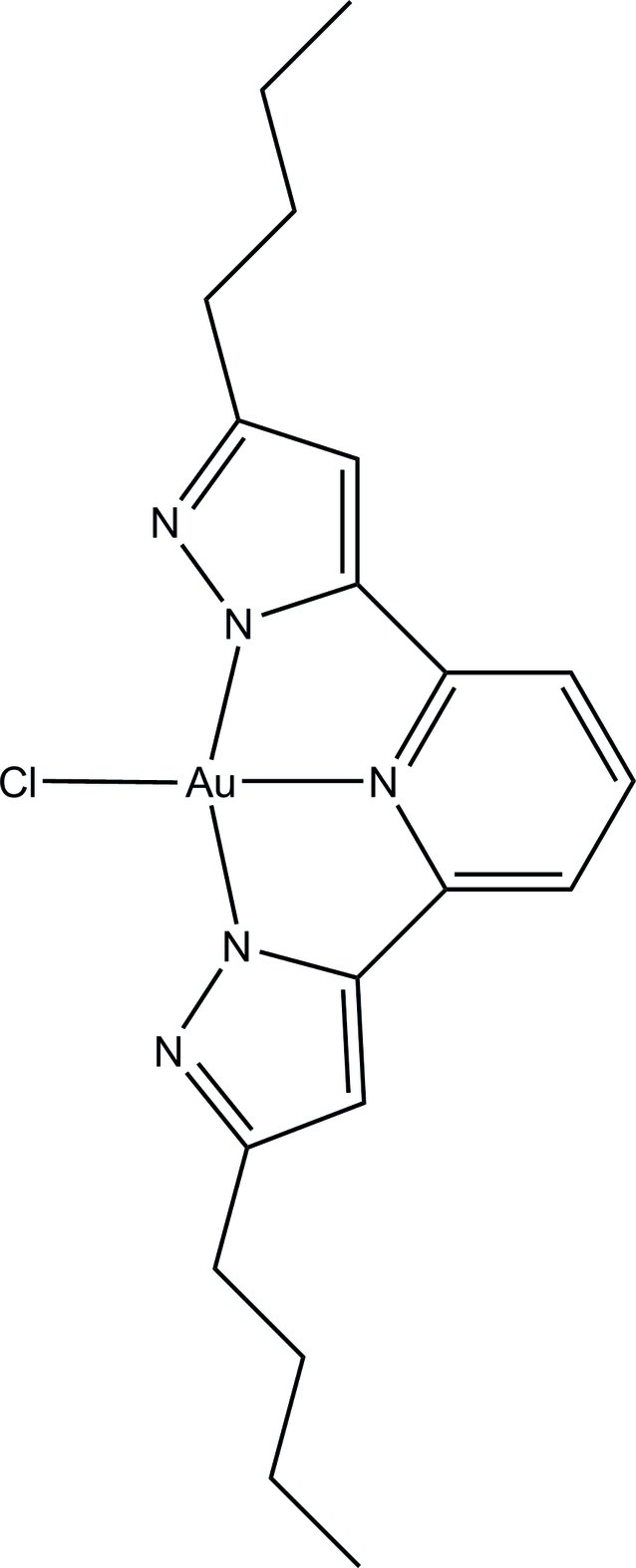


## Experimental

### 

#### Crystal data

[AuCl(C_19_H_23_N_5_)]
*M* *_r_* = 553.84
Monoclinic, 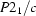

*a* = 9.0003 (3) Å
*b* = 24.2220 (7) Å
*c* = 9.3042 (3) Å
β = 101.372 (1)°
*V* = 1988.54 (11) Å^3^
*Z* = 4
Mo *K*α radiation
μ = 7.55 mm^−1^
*T* = 200 K
0.16 × 0.04 × 0.04 mm

#### Data collection

Bruker SMART CCD diffractometer
Absorption correction: multi-scan *SADABS* (Sheldrick, 2008*b* ▶) *T*_min_ = 0.378, *T*_max_ = 0.752
19560 measured reflections
4539 independent reflections
3103 reflections with *I* > 2σ(*I*)
*R*_int_ = 0.091

#### Refinement

*R*[*F* ^2^ > 2σ(*F* ^2^)] = 0.048
*wR*(*F* ^2^) = 0.089
*S* = 1.07
4539 reflections
235 parameters
H-atom parameters constrained
Δρ_max_ = 0.91 e Å^−3^
Δρ_min_ = −1.01 e Å^−3^

### 

Data collection: *SMART* (Siemens, 1996 ▶); cell refinement: *SAINT* (Siemens, 1996 ▶); data reduction: *SAINT*; program(s) used to solve structure: *SHELXTL* (Sheldrick, 2008*a* ▶); program(s) used to refine structure: *SHELXTL*; molecular graphics: *SHELXTL*; software used to prepare material for publication: *SHELXTL*.

## Supplementary Material

Crystal structure: contains datablocks I, global. DOI: 10.1107/S1600536809050995/hg2593sup1.cif

Structure factors: contains datablocks I. DOI: 10.1107/S1600536809050995/hg2593Isup2.hkl

Additional supplementary materials:  crystallographic information; 3D view; checkCIF report

## Figures and Tables

**Fig. 1.**
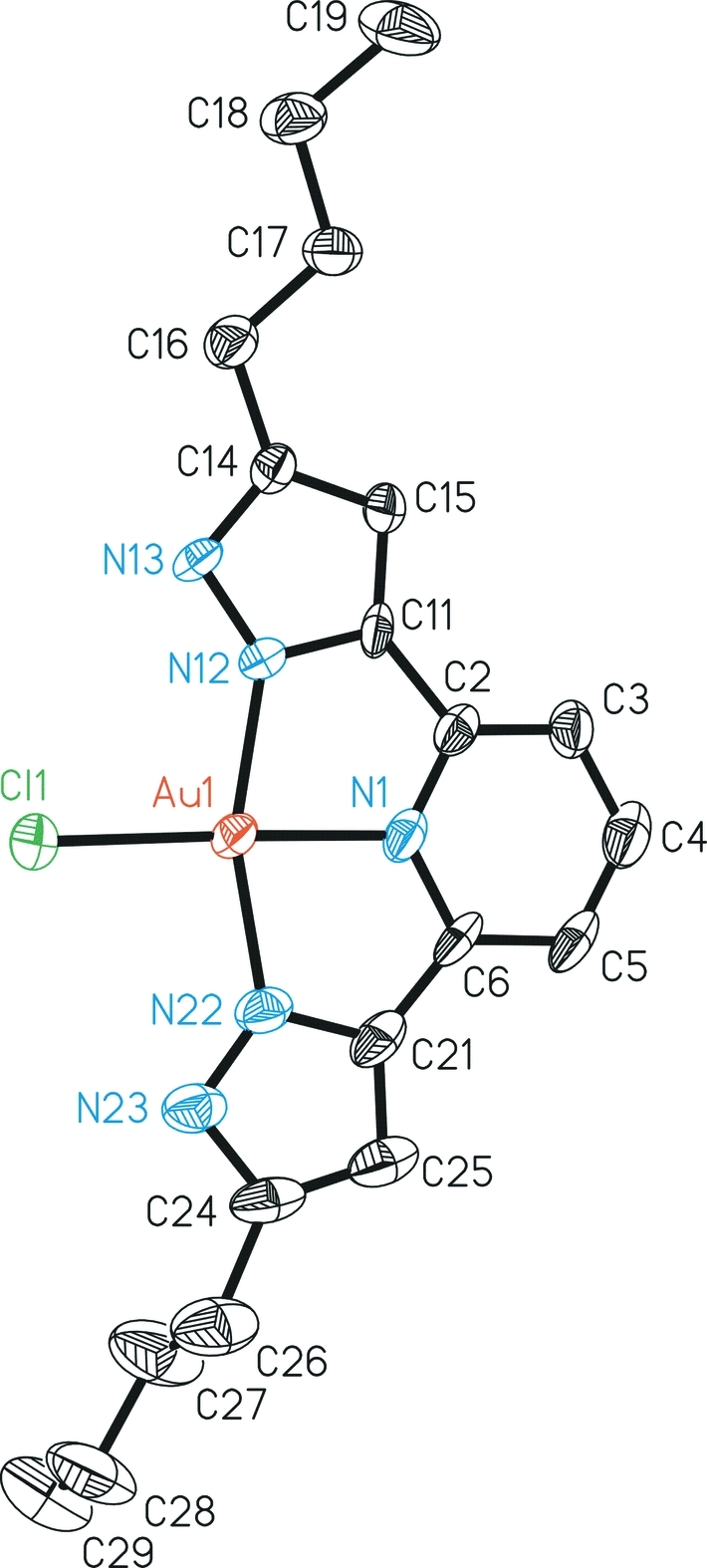
Thermal ellipsoid representation of the title compound. Displacement ellipsoids were plotted at 50% probability level.

## supplementary crystallographic information

### Comment

Catalysis of organic reactions by gold complexes has become a very important area of research in the past decade (Hashmi & Hutchings, 2006*a*, 2006*b*; Hashmi, 2007). While the field is dominated by gold(I) complexes (Ito *et al.*, 1986), the use of gold(III) pre-catalysts is also of interest (Hashmi *et al.*, 2004*a*, 2004*b*). Here we report the structural details of a new representative of the gold(III) pre-catalysts. The main feature of this structure is the ring strain of the two 5-membered metallacycles that were built up by the pincer ligand and the gold center. The theoretical sum of the bond angles in these flat 5-membered metallacycles is 540.0°, as it was observed in both cases. For a hypothetic strain-free (ring-opened) molecule simple geometrical considerations result an angle sum of about 582° (90° at Au1, 120° at N1, C2, C6, and 126° at C11, N12 C21, N22). The required adaption of 42° is achieved by bending the bond angles (mean values over both rings) at Au1 (9.7°), N1 (2.4°), C2/C6 (7.6°), C11/C21 (10.3°), and N12/N22 (12.0°). As expected the bending at the pyridin nitrogen atom N1 is by far least, as the aromatic ring itself is rigid and cannot bend on two sides simultaneously.

### Experimental

2,6-bis(5-butyl-1*H*-pyrazol-3-yl)pyridine (200 mg, µmol) was dissolved in acetone (5 ml). After this HAuCl_4_*xH_2_O (248 mg, 618 µmol, 49% metal content) in acetonitrile (3 ml) and NaOH (2.5 *M* in H_2_O, 741 µl) were added consecutively. The mixture was warmed to 60 °C for 20 min, during this time the initially formed yellow precipitate dissolved. The mixture was subjected to hot filtration and the solvent was removed under reduced pressure. The crude complex was purified by recrystallization from acetone to yield the title compound as red crystals (96.0 mg, 173 µmol, 28%). The compound is stable at RT in air.

^1^H NMR (300 MHz, acetone): δ=0.93 (t, J=7.3 Hz, 6H, CH_3_), 1.4 (dm, J=8.5, 7.1 Hz, 4H, CH_2_), 1.65 (m, 4H, CH_2_), 2.66 (t, J=7.5 Hz, 4H, CH_2_), 7.71 (d, J=7.9 Hz, 2H, ArH), 8.25 (t, J=7.9 Hz, 1H, ArH); ^13^C NMR (75 MHz, acetone): δ = 14.27, 23.13, 32.87, 106.97, 116.38 (no further signals observed, one signal overlapping with solvent at about 29 p.p.m.)

### Refinement

Carbon-bound H-atoms were placed in calculated positions (C–H 0.95– 0.99 Å) and were included in the refinement in the riding model approximation with *U*_iso_(H) set to 1.2–1.5*U*_eq_(C). A staggered group model was used for the methyl groups.

### Crystal data

**Table:**

[AuCl(C_19_H_23_N_5_)]	*Z* = 4
*M**_r_* = 553.84	*F*(000) = 1072
Monoclinic, *P*2_1_/*c*	*D*_x_ = 1.850 Mg m^−^^3^
Hall symbol: -P 2ybc	Mo *K*α radiation, λ = 0.71073 Å
*a* = 9.0003 (3) Å	Cell parameters from 6701 reflections
*b* = 24.2220 (7) Å	µ = 7.55 mm^−^^1^
*c* = 9.3042 (3) Å	*T* = 200 K
β = 101.372 (1)°	Polyhedron, orange
*V* = 1988.54 (11) Å^3^	0.16 × 0.04 × 0.04 mm

### Data collection

**Table:**

Bruker SMART CCD diffractometer	4539 independent reflections
Radiation source: fine-focus sealed tube	3103 reflections with *I* > 2σ(*I*)
graphite	*R*_int_ = 0.091
ω scans	θ_max_ = 27.5°, θ_min_ = 1.7°
Absorption correction: multi-scan *SADABS* (Sheldrick, 2008b)	*h* = −11→11
*T*_min_ = 0.378, *T*_max_ = 0.752	*k* = −31→31
19560 measured reflections	*l* = −12→12

### Refinement

**Table:**

Refinement on *F*^2^	Primary atom site location: structure-invariant direct methods
Least-squares matrix: full	Secondary atom site location: difference Fourier map
*R*[*F*^2^ > 2σ(*F*^2^)] = 0.048	Hydrogen site location: inferred from neighbouring sites
*wR*(*F*^2^) = 0.089	H-atom parameters constrained
*S* = 1.07	*w* = 1/[σ^2^(*F*_o_^2^) + (0.0241*P*)^2^ + 2.5681*P*] where *P* = (*F*_o_^2^ + 2*F*_c_^2^)/3
4539 reflections	(Δ/σ)_max_ = 0.001
235 parameters	Δρ_max_ = 0.91 e Å^−^^3^
0 restraints	Δρ_min_ = −1.01 e Å^−^^3^

### Special details

**Table:**

Geometry. All e.s.d.'s (except the e.s.d. in the dihedral angle between two l.s. planes) are estimated using the full covariance matrix. The cell e.s.d.'s are taken into account individually in the estimation of e.s.d.'s in distances, angles and torsion angles; correlations between e.s.d.'s in cell parameters are only used when they are defined by crystal symmetry. An approximate (isotropic) treatment of cell e.s.d.'s is used for estimating e.s.d.'s involving l.s. planes.
Refinement. Refinement of *F*^2^ against ALL reflections. The weighted *R*-factor *wR* and goodness of fit *S* are based on *F*^2^, conventional *R*-factors *R* are based on *F*, with *F* set to zero for negative *F*^2^. The threshold expression of *F*^2^ > σ(*F*^2^) is used only for calculating *R*-factors(gt) *etc*. and is not relevant to the choice of reflections for refinement. *R*-factors based on *F*^2^ are statistically about twice as large as those based on *F*, and *R*- factors based on ALL data will be even larger.

### Fractional atomic coordinates and isotropic or equivalent isotropic displacement parameters (Å^2^)

**Table:**

	*x*	*y*	*z*	*U*_iso_*/*U*_eq_	
Au1	0.28448 (3)	0.481560 (12)	0.61190 (3)	0.03110 (10)	
Cl1	0.3449 (3)	0.44522 (9)	0.8400 (2)	0.0514 (6)	
N1	0.2300 (7)	0.5101 (2)	0.4082 (6)	0.0347 (15)	
C2	0.1273 (8)	0.5513 (3)	0.3826 (8)	0.0328 (17)	
C3	0.0815 (9)	0.5696 (3)	0.2393 (8)	0.040 (2)	
H3	0.0069	0.5977	0.2156	0.048*	
C4	0.1478 (11)	0.5458 (3)	0.1317 (9)	0.050 (2)	
H4	0.1166	0.5578	0.0331	0.060*	
C5	0.2585 (10)	0.5050 (3)	0.1635 (9)	0.044 (2)	
H5	0.3070	0.4906	0.0897	0.053*	
C6	0.2952 (8)	0.4865 (3)	0.3063 (8)	0.0344 (18)	
C11	0.0767 (8)	0.5693 (3)	0.5158 (7)	0.0316 (18)	
N12	0.1375 (7)	0.5411 (2)	0.6419 (6)	0.0319 (14)	
N13	0.0917 (6)	0.5633 (2)	0.7583 (6)	0.0293 (14)	
C14	−0.0014 (8)	0.6050 (3)	0.7037 (8)	0.0311 (17)	
C15	−0.0165 (8)	0.6099 (3)	0.5537 (8)	0.0328 (18)	
H15	−0.0773	0.6355	0.4906	0.039*	
C16	−0.0734 (9)	0.6395 (3)	0.8036 (8)	0.040 (2)	
H16A	0.0009	0.6458	0.8958	0.048*	
H16B	−0.1601	0.6190	0.8284	0.048*	
C17	−0.1289 (9)	0.6948 (3)	0.7383 (9)	0.042 (2)	
H17A	−0.2064	0.6882	0.6485	0.050*	
H17B	−0.0429	0.7142	0.7085	0.050*	
C18	−0.1964 (9)	0.7326 (3)	0.8389 (9)	0.048 (2)	
H18A	−0.1224	0.7370	0.9322	0.057*	
H18B	−0.2881	0.7149	0.8618	0.057*	
C19	−0.2390 (11)	0.7898 (4)	0.7739 (11)	0.066 (3)	
H19A	−0.1466	0.8108	0.7709	0.098*	
H19B	−0.2990	0.8094	0.8349	0.098*	
H19C	−0.2986	0.7858	0.6743	0.098*	
C21	0.3986 (9)	0.4418 (3)	0.3670 (8)	0.041 (2)	
N22	0.4106 (7)	0.4319 (3)	0.5137 (7)	0.0386 (16)	
N23	0.5062 (8)	0.3898 (3)	0.5598 (8)	0.0465 (18)	
C24	0.5559 (9)	0.3730 (3)	0.4398 (11)	0.049 (2)	
C25	0.4926 (9)	0.4040 (3)	0.3161 (10)	0.049 (2)	
H25	0.5099	0.4002	0.2191	0.058*	
C26	0.6668 (11)	0.3245 (4)	0.4558 (13)	0.071 (3)	
H26A	0.7227	0.3261	0.3744	0.085*	
H26B	0.7416	0.3291	0.5484	0.085*	
C27	0.6011 (12)	0.2719 (4)	0.4563 (15)	0.093 (4)	
H27A	0.5352	0.2656	0.3592	0.112*	
H27B	0.5353	0.2718	0.5299	0.112*	
C28	0.7134 (12)	0.2229 (5)	0.4898 (16)	0.094 (4)	
H28A	0.7856	0.2244	0.4221	0.113*	
H28B	0.7723	0.2265	0.5911	0.113*	
C29	0.6347 (14)	0.1689 (5)	0.4739 (15)	0.114 (5)	
H29A	0.5526	0.1693	0.5293	0.171*	
H29B	0.7069	0.1396	0.5119	0.171*	
H29C	0.5924	0.1620	0.3701	0.171*	

### Atomic displacement parameters (Å^2^)

**Table:**

	*U*^11^	*U*^22^	*U*^33^	*U*^12^	*U*^13^	*U*^23^
Au1	0.03813 (17)	0.03202 (16)	0.02527 (15)	0.00177 (16)	0.01141 (11)	−0.00274 (16)
Cl1	0.0686 (15)	0.0563 (14)	0.0304 (11)	0.0218 (12)	0.0125 (10)	0.0075 (10)
N1	0.051 (4)	0.032 (4)	0.024 (3)	−0.006 (3)	0.015 (3)	−0.003 (3)
C2	0.039 (5)	0.034 (4)	0.029 (4)	−0.008 (4)	0.016 (3)	−0.001 (3)
C3	0.053 (5)	0.040 (5)	0.026 (4)	0.001 (4)	0.005 (4)	0.005 (4)
C4	0.079 (7)	0.041 (5)	0.032 (5)	−0.014 (5)	0.019 (5)	−0.003 (4)
C5	0.072 (6)	0.039 (5)	0.030 (4)	−0.021 (4)	0.031 (4)	−0.012 (4)
C6	0.045 (5)	0.040 (5)	0.024 (4)	−0.014 (4)	0.021 (3)	−0.010 (4)
C11	0.043 (5)	0.034 (4)	0.019 (4)	−0.003 (3)	0.010 (3)	0.008 (3)
N12	0.038 (4)	0.033 (3)	0.027 (3)	0.007 (3)	0.013 (3)	−0.002 (3)
N13	0.029 (3)	0.039 (4)	0.022 (3)	0.001 (3)	0.010 (3)	−0.008 (3)
C14	0.032 (4)	0.037 (5)	0.024 (4)	0.000 (3)	0.007 (3)	0.000 (3)
C15	0.041 (5)	0.035 (4)	0.021 (4)	0.005 (4)	0.003 (3)	0.002 (3)
C16	0.041 (5)	0.048 (5)	0.031 (4)	0.005 (4)	0.006 (4)	−0.003 (4)
C17	0.045 (5)	0.041 (5)	0.038 (5)	0.011 (4)	0.004 (4)	−0.006 (4)
C18	0.051 (6)	0.050 (5)	0.039 (5)	0.009 (4)	0.003 (4)	−0.014 (4)
C19	0.080 (7)	0.042 (6)	0.067 (7)	0.018 (5)	−0.005 (5)	−0.018 (5)
C21	0.054 (5)	0.041 (5)	0.031 (5)	−0.012 (4)	0.020 (4)	−0.014 (4)
N22	0.046 (4)	0.032 (4)	0.041 (4)	0.002 (3)	0.018 (3)	−0.007 (3)
N23	0.047 (4)	0.034 (4)	0.067 (5)	0.001 (3)	0.031 (4)	0.001 (4)
C24	0.046 (5)	0.031 (5)	0.079 (7)	−0.007 (4)	0.036 (5)	−0.016 (5)
C25	0.050 (6)	0.040 (5)	0.064 (6)	−0.007 (4)	0.031 (5)	−0.016 (5)
C26	0.075 (7)	0.041 (6)	0.106 (9)	−0.002 (5)	0.041 (6)	−0.018 (6)
C27	0.055 (7)	0.064 (8)	0.170 (13)	0.009 (6)	0.042 (7)	0.025 (8)
C28	0.062 (7)	0.071 (9)	0.152 (12)	0.032 (6)	0.027 (7)	0.030 (8)
C29	0.104 (11)	0.092 (11)	0.141 (13)	0.044 (9)	0.011 (9)	0.018 (10)

### Geometric parameters (Å, °)

**Table:**

Au1—N1	1.985 (6)	C17—H17B	0.9900
Au1—N22	1.994 (6)	C18—C19	1.529 (11)
Au1—N12	2.014 (6)	C18—H18A	0.9900
Au1—Cl1	2.263 (2)	C18—H18B	0.9900
N1—C6	1.338 (8)	C19—H19A	0.9800
N1—C2	1.349 (9)	C19—H19B	0.9800
C2—C3	1.389 (10)	C19—H19C	0.9800
C2—C11	1.469 (9)	C21—N22	1.369 (9)
C3—C4	1.387 (11)	C21—C25	1.390 (11)
C3—H3	0.9500	N22—N23	1.349 (9)
C4—C5	1.394 (12)	N23—C24	1.346 (10)
C4—H4	0.9500	C24—C25	1.398 (12)
C5—C6	1.379 (10)	C24—C26	1.530 (12)
C5—H5	0.9500	C25—H25	0.9500
C6—C21	1.466 (11)	C26—C27	1.403 (12)
C11—N12	1.374 (9)	C26—H26A	0.9900
C11—C15	1.383 (10)	C26—H26B	0.9900
N12—N13	1.345 (7)	C27—C28	1.551 (13)
N13—C14	1.346 (9)	C27—H27A	0.9900
C14—C15	1.381 (9)	C27—H27B	0.9900
C14—C16	1.490 (10)	C28—C29	1.482 (15)
C15—H15	0.9500	C28—H28A	0.9900
C16—C17	1.515 (10)	C28—H28B	0.9900
C16—H16A	0.9900	C29—H29A	0.9800
C16—H16B	0.9900	C29—H29B	0.9800
C17—C18	1.519 (10)	C29—H29C	0.9800
C17—H17A	0.9900		
			
N1—Au1—N22	80.1 (3)	C17—C18—C19	113.6 (7)
N1—Au1—N12	80.6 (2)	C17—C18—H18A	108.8
N22—Au1—N12	160.8 (3)	C19—C18—H18A	108.8
N1—Au1—Cl1	177.47 (18)	C17—C18—H18B	108.8
N22—Au1—Cl1	98.2 (2)	C19—C18—H18B	108.8
N12—Au1—Cl1	101.06 (18)	H18A—C18—H18B	107.7
C6—N1—C2	124.9 (6)	C18—C19—H19A	109.5
C6—N1—Au1	117.9 (5)	C18—C19—H19B	109.5
C2—N1—Au1	117.3 (5)	H19A—C19—H19B	109.5
N1—C2—C3	118.0 (7)	C18—C19—H19C	109.5
N1—C2—C11	112.7 (6)	H19A—C19—H19C	109.5
C3—C2—C11	129.2 (7)	H19B—C19—H19C	109.5
C4—C3—C2	118.1 (8)	N22—C21—C25	106.9 (8)
C4—C3—H3	120.9	N22—C21—C6	115.6 (6)
C2—C3—H3	120.9	C25—C21—C6	137.5 (8)
C3—C4—C5	122.2 (8)	N23—N22—C21	111.6 (6)
C3—C4—H4	118.9	N23—N22—Au1	134.0 (5)
C5—C4—H4	118.9	C21—N22—Au1	114.3 (5)
C6—C5—C4	117.5 (7)	C24—N23—N22	105.1 (7)
C6—C5—H5	121.3	N23—C24—C25	111.9 (7)
C4—C5—H5	121.3	N23—C24—C26	117.9 (9)
N1—C6—C5	119.2 (8)	C25—C24—C26	130.2 (8)
N1—C6—C21	112.1 (6)	C21—C25—C24	104.5 (8)
C5—C6—C21	128.7 (7)	C21—C25—H25	127.8
N12—C11—C15	107.2 (6)	C24—C25—H25	127.8
N12—C11—C2	115.8 (7)	C27—C26—C24	115.5 (9)
C15—C11—C2	137.0 (7)	C27—C26—H26A	108.4
N13—N12—C11	110.8 (6)	C24—C26—H26A	108.4
N13—N12—Au1	135.4 (5)	C27—C26—H26B	108.4
C11—N12—Au1	113.6 (5)	C24—C26—H26B	108.4
N12—N13—C14	105.2 (5)	H26A—C26—H26B	107.5
N13—C14—C15	112.1 (6)	C26—C27—C28	115.9 (9)
N13—C14—C16	120.0 (6)	C26—C27—H27A	108.3
C15—C14—C16	127.9 (7)	C28—C27—H27A	108.3
C14—C15—C11	104.6 (6)	C26—C27—H27B	108.3
C14—C15—H15	127.7	C28—C27—H27B	108.3
C11—C15—H15	127.7	H27A—C27—H27B	107.4
C14—C16—C17	113.3 (6)	C29—C28—C27	112.1 (9)
C14—C16—H16A	108.9	C29—C28—H28A	109.2
C17—C16—H16A	108.9	C27—C28—H28A	109.2
C14—C16—H16B	108.9	C29—C28—H28B	109.2
C17—C16—H16B	108.9	C27—C28—H28B	109.2
H16A—C16—H16B	107.7	H28A—C28—H28B	107.9
C16—C17—C18	115.2 (7)	C28—C29—H29A	109.5
C16—C17—H17A	108.5	C28—C29—H29B	109.5
C18—C17—H17A	108.5	H29A—C29—H29B	109.5
C16—C17—H17B	108.5	C28—C29—H29C	109.5
C18—C17—H17B	108.5	H29A—C29—H29C	109.5
H17A—C17—H17B	107.5	H29B—C29—H29C	109.5
